# Sustainable development of environmental protection talents training: Research on the behavior decision of government, university and enterprise under the background of evolutionary game

**DOI:** 10.1371/journal.pone.0298548

**Published:** 2024-02-23

**Authors:** Jinxia Wang, Yunfeng Tan, Lingling Zhan, Hongjun Yang, Xieling Li, Fang Gao, Siyuan Qiu

**Affiliations:** 1 College of Resources and Safety, Chongqing Vocational Institute of Engineering, Chongqing, China; 2 College of River and Ocean Engineering, Chongqing Jiaotong University, Chongqing, China; 3 General college, Chongqing Vocational Institute of Engineering, Chongqing, China; 4 College of Resources and Environment, Southwest University, Beibei, Chongqing, China; Harbin Institute of Technology, CHINA

## Abstract

Environmental protection talents training (EPTT) is recognized as a key prerequisite for maintaining environmental sustainability, and in order to study the influence of each player on EPTT. This paper innovatively constructs a tripartite evolutionary game model of government, university and enterprise. The equilibrium points and evolutionary stabilization strategies of each participant are solved by replicating the dynamic equations, and the behaviors of each subject in EPTT are analyzed so as to clarify the behavioral characteristics and optimal strategies of the government’s participation in EPTT. The results show that enterprises occupy a more important position in influencing government decisions. The government should reduce the financial incentives for enterprises and replace them with greater policy support. Meanwhile, the government should actively promote the cultivation mechanism that integrates universities and enterprises. The results of the study can provide a decision-making basis for the government to promote the sustainable development of EPTT.

## 1 Introduction

Environmental pollution has become one of the major challenges to the sustainable development of human society [1–3]. According to statistics released by the United Nations Environment Programme (UNEP), the amount of waste alone generated worldwide is about approximately 7–10 billion tons of waste per year [4]. This waste causes environmental pollution, depletion of natural resources and climate change [5, 6]. In addition to this, environmental pollution of air, water and soil poses a huge and growing threat to the health of the global ecosystem [7–9]. According to The Lancet Commission on Pollution and Health, environmental pollution is the biggest cause of disease and premature death in the world today [10]. Statistics show that pollution-induced diseases are responsible for an estimated 9 million premature deaths, or 16% of all deaths globally, and three times as many as AIDS, malaria and tuberculosis combined [11]. More than half of the world ’s population lacks access to safe drinking water [12]. Although China is one of the poorest countries in terms of water resources per capita, it is the world ’s largest country in terms of sewage discharges [13]. Solid waste generation also ranks first among the world’s five largest economies [14, 15]. What’s more, China has declared to peak its carbon emissions by 2030 as a nationally owned contribution and to further achieve carbon neutrality by 2060 [16–18]. This also puts forward higher requirements for China’s environmental protection endeavors. Therefore, Therefore, how to deal with the relationship between economic development and environmental protection reasonably and effectively, so that the environment is not polluted and destroyed, is undoubtedly an important issue that the China government needs to consider urgently [19, 20].

Industrial production boosts economic growth, but also produces large amounts of pollutants, while putting enormous pressure on the environment [21, 22]. Many industrial chemicals are chemically and physiologically stable and can persist in the environment and biological systems [23]. They are also highly lipophilic, which leads to their bioaccumulation in the fatty tissues of animals and humans [24]. Environmental protection in industrial production processes has therefore received widespread attention around the world. China is the world ’s largest emerging economy, has entered the advanced stage of industrialization [25]. At this time, more manufacturing enterprises and production enterprises are facing industrial upgrading, which requires enterprises to explore in the fields of green innovation and technological innovation [26, 27]. Enterprises (EP) are more likely to strengthen pollution control and management in the production process, while the state should also vigorously pursue sustainable economic and environmental development [28, 29]. In recent years, low-carbon and digitalization have become the general trend of manufacturing upgrading and transformation. Digital technology empowers the whole process of green manufacturing and realizes the coordinated and unified development of economic development and environmental protection [30, 31]. These new technologies are also widely used in the fields of machinery manufacturing, rural development and revitalization. In recent years, with the advancement of the dual carbon goal and the rural revitalization strategy, the development of new rural energy has a new direction [32, 33]. Therefore a large number of high-quality, high-skilled and high-level environmentally friendly professionals are essential [34–36]. They mainly include talents in ecological environment monitoring, environmental governance, environmental planning, and ecological environment law enforcement [37, 38]. The cultivation of these environmental protection talents (EPTT) has become an important prerequisite for realizing the management of environmental problems and sustainable development [39, 40]. In addition, the important task of environmental protection requires the supervision and leadership of the state and the government (GM) [41, 42]. Recognizing the importance of this phenomenon, China’s GM has formulated multiple policies for the training of talents in ecological environmental protection. By the end of 2020, the total number of ecological and environmental protection talents in China reached approximately 243,000, an increase of 35.8% compared to 2010. Among them, the proportion of talents with higher education and senior professional titles has been increasing year by year. The number of talents with a master’s degree or above is approximately 26,000, an increase of 120%; and the number of talents with senior professional titles is approximately 18,000, an increase of 50%. However, the current ecological and environmental protection talent pool in China still cannot meet the needs of the new ecological and environmental protection situation in terms of quantity, structure, and institutional policies [37, 43]. In conclusion, EPTT is an important prerequisite for solving environmental problems and requires the joint participation of GM, EP, universities (US) and other parties.

**Fig 1:**
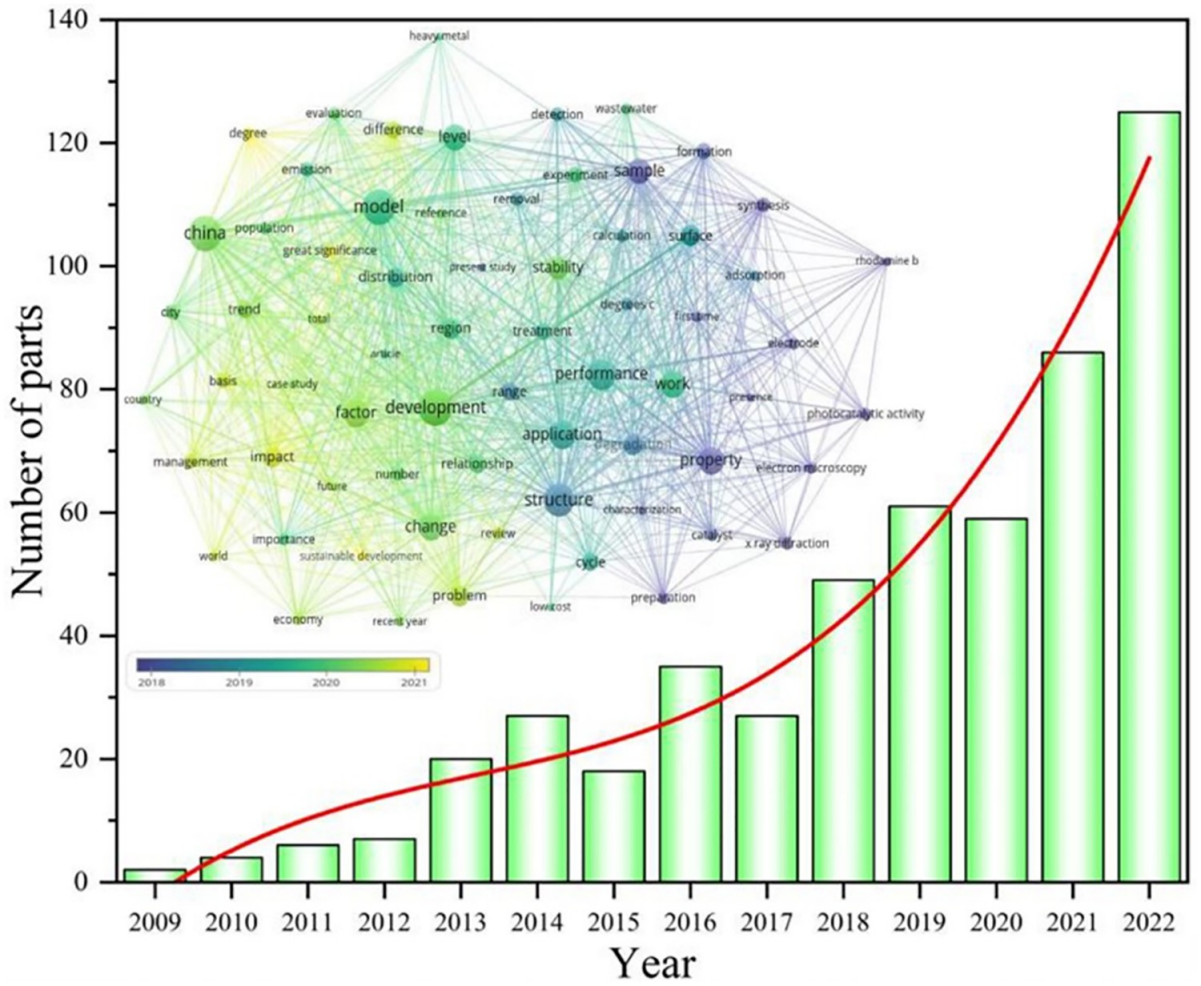
Statistical map of scientometrics academic research results (Academic papers were searched based on the keywords "education" and "environmentally friendly talents". The data were analyzed using the keyword co-occurrence function built into VOSviewer and plotted in the "Network Visualization" and "Overlay Visualization (Years)"; the bar graphs represent the number of publications).

In recent years, countries all over the world are making efforts to formulate policies and actively exploring effective implementation measures, including in the education system. As a result, more and more scholars are shifting their focus to EPTT. Fig 1 shows the statistical chart of academic research results in scientometrics. This graph depicts the hotspots analysis of EPTT from 2018 to 2022. It can be seen that EPTT is widely regarded, involving many countries, regions, and cities. Among them, China’s attention is especially prominent. EPTT focuses on the treatment of wastewater, heavy metal pollution and other fields. At the same time, environmental material performance identification, structural analysis and application have also become the focus of research [44, 45]. In addition, the development trend of environmental sustainability and modeling prediction are also included. Through the statistics of the number of publications, it can be seen that it is mainly divided into two phases, the first phase in 2009–2015. It is characterized by a slow growth period, while the second stage is a period of rapid growth in 2016–2012, which also reflects the continuous growth of people’s attention to EPTT.

The strict formulation and implementation of environmental policies are beneficial safeguards for environmental protection [46]. However, improper waste management policy will not only lead to waste of resources and economic development, but also bring heavy financial burden to the government. This will pose a serious challenge and threat to environmental sustainability and human health. EP must be equipped with professional environmental protection talents in order to effectively cope with the problem of environmental impacts brought about by the development of EP, which is also the EP’s implementation of cleaner production to achieve the goal of sustainable This is also an important prerequisite for EP to implement cleaner production and realize sustainable goals. However, the diversity of industrial types in the country increases the complexity and uncertainty of environmental protection due to the complexity of EP forms. Therefore, it is crucial to cultivate environmental protection talents that meet the diverse pollution control and management requirements of EP. Some scholars believe that US should take the key responsibility of EPTT, and EP is also needed to provide practical platforms and practical cases for EPTT to help students better understand and apply what they have learned about environmental protection. Both US and EP have irreplaceable roles in EPTT. They are jointly advancing the development of environmental protection cause. US and EP need to merge in EPTT, which contributes to the precision of EPTT. Therefore, the EPTT process is very complex, and the main stakeholders involved in EPTT can be divided into three aspects: namely GM, US, and EP.

As a result, many scholars have conducted research on the cultivation of environmental talents, and some scholars have studied the role of human capital in green growth and environmental performance, which has shown that increased investment in human capital can promote green economic growth and realize environmental protection [47]. Yang et al. [48] empirically analyzed the impact of natural disasters on agricultural production decisions using the probit model. The results of this study can provide empirical evidence for the government to formulate relevant policies. Sohail et al. [49] investigated the relationship between the educational level of agricultural workers and their response to environmental risks by means of PLS-SEM statistical analysis, and the study showed that education may, to some extent, influence farmers’ strategies for adapting to environmental change. Some scholars have also analyzed the asymmetric effect of economic policy uncertainty on green growth in highly polluting economies over the period 1994–2020 through the CS-ARDL model, suggesting that the government must consider the issue of economic policy uncertainty when formulating green growth policies [50]. In addition, a variety of modeling research theories have emerged to study the interactions between multiple subjects. For example, Yu et al. [51] analyzed the new energy enterprise-village collective linkage through the model of quantum entanglement and benefit relationship, and their study also considered the characteristics of quantum entanglement in the implementation of the quantum game. Yin et al. [52] analyzed the stochastic differential game of low carbon technology sharing in the collaborative innovation system between advantaged and disadvantaged enterprises in an uncertain environment. Some scholars [53] have also analyzed the behaviors and strategies of three key stakeholders, namely, the government, construction contractors and waste recycling plants, in promoting the sustainable development of the C&D waste recycling market through evolutionary game theory, and provided valuable management suggestions for the government. Although scholars have made important research results on model construction and application. These theoretical findings have also enriched the theoretical and applied foundations of multi-subject research. However, the gaps in the existing research lie in the fact that there are fewer studies involving the decision-making behaviors of the government and enterprises in EPTT, and there is a lack of clear research results on the behavioral and decision-making characteristics of multi-subjects as well as the game relationship between subjects. At the same time, there is a greater lack of exploration of the feasibility of EP participation in EPTT in China. Therefore, it is necessary to take EPs as the stakeholders of the game and observe the influence of various factors on the behavioral decisions and overall operational effects of each stakeholder in EPTT.

By constructing a three-party evolutionary game model of GM, US and EP, the influencing factors of EPTT are identified, and the attitudes and behaviors of stakeholders in EPTT are examined. Compared with previous studies, this paper applies evolutionary game theory to talent cultivation, expands the application scope of evolutionary game theory, and considers the decision-making behavior of EP, which provides a promising new idea for the future research of evolutionary game theory and EPTT. The decision-making characteristics of tripartite behavior under initial policy conditions and the evolution trend of tripartite behavior after policy conditions change are evaluated. The model is developed based on rigorous mathematical analysis, which is more realistic and objective in determining the decision-making behaviors of the stakeholders in EPTT, and is conducive to the search for effective policy and management solutions to enhance the overall operational effectiveness of EPTT in China.

## 2 Evolutionary game modeling

### 2.1 Game subject analyzer hypothesis proposed

**Fig 2:**
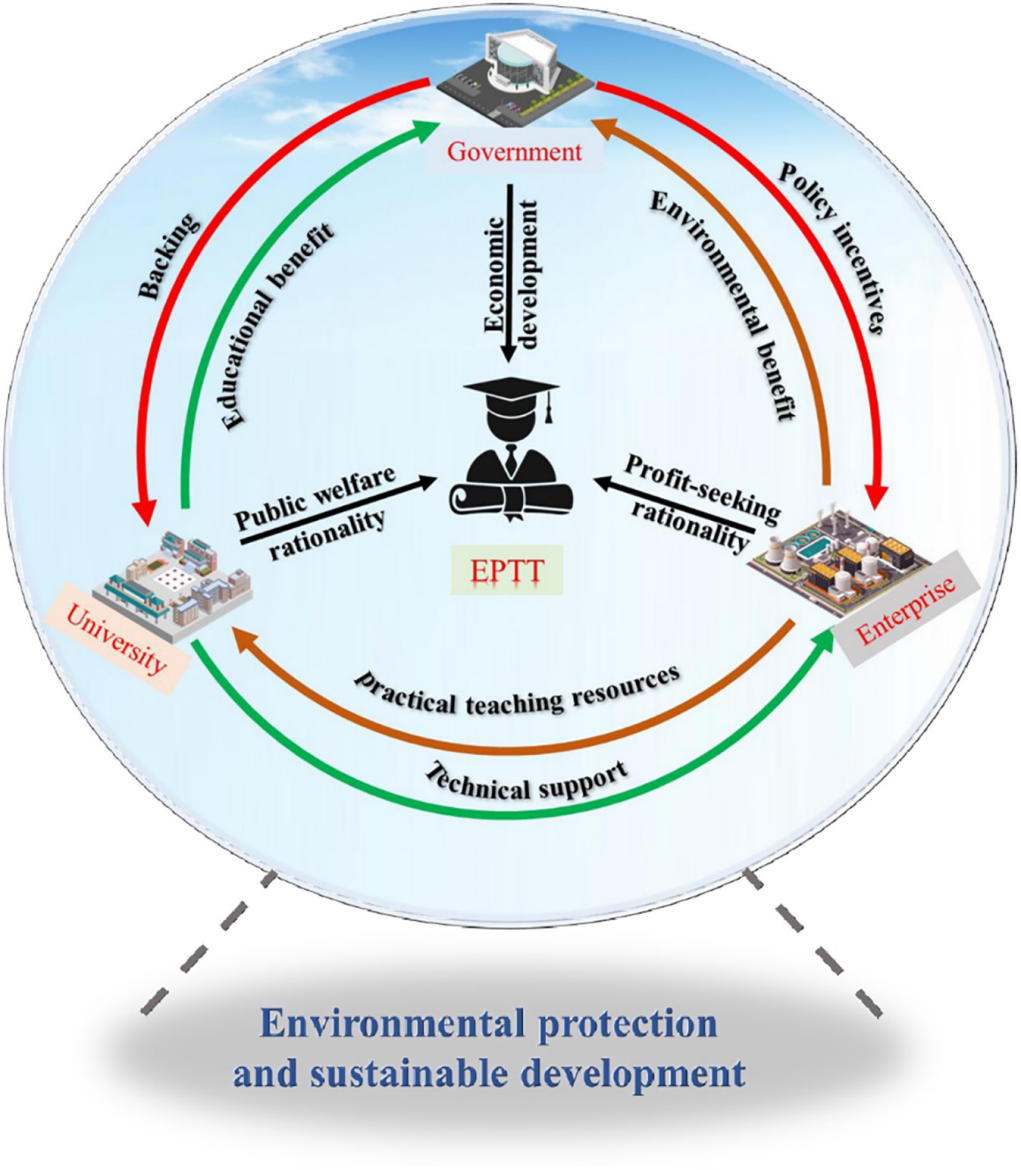
Stakeholder game framework for EPTT.

Before constructing the three-way evolutionary game model, the stakeholders and game rules in the system need to be identified. Fig 2 depicts the relationships and game rules among the three stakeholders in the EPTT system. Regarding EPTT, the GM, US, and EP are key stakeholders, each playing the roles of promoter, implementer, and executor. Based on the introduction of the current situation and related issues in China, the GM is responsible for developing EPTT guidelines and regulations, providing financial support to US EPTT, and offering additional incentives to the US when they actively implement.

At the same time, GM establishes a monitoring mechanism for EPTT and penalizes EPs when they do not implement EPTT to improve the quality of EPTT. When EPs actively implement it, GM gives incentives to EPs to increase the motivation of EPs to participate in EPTT. On the other hand, EP can receive rewards from GM, actively implement EPTT, and gain reputation benefits, bringing environmental benefits to GM. Therefore, GM can obtain educational and environmental benefits when promoting EPTT.

To sum up, GM, US and EP are three key stakeholders in the system, and the strategy of any one will affect the strategy of the other two. In the following, a three-party evolutionary game model will be constructed based on their strategies. In order to explain the model more clearly, this paper makes the following assumptions in light of the real situation:

Assumption 1: This game involves three participants: GM, HE and EP, and all three parties are finite rationality. GM’s behavioral strategy set S_1_ = (incentives, disincentives), "incentives" means that GM invests human resources, material resources, financial resources, etc. to support US’s EPTT, and rewards the environmental protection EP’s participation in the EPTT; GM’s The "disincentive" strategy is defined as the GM’s policy support for EPTT without financial support or incentives to US and EPs. It mainly consists of GM no longer penalizing EPs for "negative implementation". The set of US behavioral strategies S_2_ = (positive implementation, negative implementation), with "positive implementation" meaning that the US actively promotes the EPTT, and "negative implementation" meaning that the US actively promotes the EPTT despite setting up the EPTT. "Positive implementation" means that the US sets up teaching facilities for EPTT but does not actively promote it. EP’s behavioral strategy set S_3_ = (positive implementation, negative implementation), "Positive implementation" means that the EP strictly follows the GM’s requirements, sets up environmental positions and receives environmental personnel trained by the US and participates in the EPTT. "Negative implementation" means that the EP negatively implements the GM’s requirements, sets up environmental positions but does not accept the environmental talents trained by the US, adopts non-environmental professionals to undertake the EP’s environmental work, and negatively participates in the EPTT.
Assumption 2: The probability that the GM department chooses to incentivize is x(0≤x≤1) and the probability that it chooses not to incentivize is 1-x; the probability that the US chooses to implement positively is y(0≤y≤1) and the probability that it chooses to implement negatively is 1-y; the probability that the EP implements positively is z(0≤z≤1) and the probability that it chooses to implement negatively is 1-z.
Assumption 3: In order to meet the needs of EPTT, US has to invest in the construction of teaching facilities and teacher training to meet the requirements of teaching activities, and in order to ensure the quality of EPTT, US also has to seek the conditions of practical teaching from EP, and at the same time obtain the special funding given by GM for running the school.
Assumption 4: EP may pollute the environment due to its production activities, so GM formulates regulations to constrain EP by requiring it to establish environmental protection positions and hire environmental protection professionals. At the same time, EP should also assist US in implementing the practical training component of EPTT, provide US with practical teaching resources, and be penalized by GM if it fails to do so. On the contrary, if EP actively implements it, GM will give some policy support. Meanwhile, the public image of EP will be enhanced.
Assumption 5: GM develops EPTT guidelines and specifications, stipulates that US is responsible for the implementation of EPTT, and EP receives environmentally friendly personnel and cooperates with US in the EPTT process. GM incentivizes by paying fiscal and regulatory costs and pays the cost of additional incentives to EP and US. Based on the above assumptions, the relevant variables of the evolutionary game are shown in Table 1.

**Table 1:** Variables and meanings related to the three-way game.

Interested party	Income and expenditure	Variable symbol	Variable meanings
GM	expenditures	O_g1_	Costs of developing laws and regulations and implementing norms
		O_g2_	EPTT financial outlays to US
		O_g3_	Rewards for EPs when they are "actively performing"
		O_g4_	Rewards for improving overall school performance when US is "actively implemented"
	earnings	I_g1_	Public image gained by GM when US is "actively implemented"
		I_g2_	Public image gained by GM when EP is "actively implemented"
		I_g3_	Penalization of EP’s income in case of EP’s "negative enforcement"
US	expenditures	O_u1_	Construction costs of teaching facilities
		O_u2_	US Faculty Training Fees
		O_u3_	Collaborative schooling inputs between US and EPs
		α	US expenditure factor
	earnings	O_g2_	Specialized school support from GM
		O_g4_	Overall US school strength improved and awards received
		I_u3_	Improved overall strength of the US, enhanced reputation, and social benefits gained
EP	expenditures	O_e1_	EP Expenditures for the creation of environmental posts
		I_g3_	Expenditures penalized by the GM in case of EP "Negative Enforcement"
		O_e3_	Expenditures by EP in collaboration with US to provide EPTT with practical teaching conditions
		β	EP expenditure factor
	earnings	Ie_1_	Policy support from GM
		O_g3_ = I_e2_	Rewards given by the GM when the EP is "actively executed"
		I_e3_	EP image enhancement, social benefits gained

### 2.2 Model construction

Based on the above assumptions, the GM, US and EP game strategy selection leads to 8 groups of game combination strategies, and the specific benefit analysis is shown in Table 2.

**Table 2:** Combination of strategies and payoffs of the three-way game.

strategy combination	GM(x)	US(y)	EP(z)
(Motivation, active implementation, positive enforcement)	I_g1_+I_g2_-O_g1_-O_g2_-O_g3_-O_g4_	O_g2_+O_g4_+I_u3_-α(O_u1_+O_u2_+O_u3_)	I_e1_+O_g3_+I_e3_ -β(O_e1_+O_e3_)
(Incentives, positive implementation, negative enforcement)	I_g1_+I_g3_-O_g1_-O_g2_-O_g4_	O_g2_+O_g4_+I_u3_-α(O_u1_+O_u2_)	I_e1_-β(O_e1_+O_e3_)-I_g3_
(Incentives, negative implementation, positive implementation)	I_g2_-O_g1_-O_g2_-O_g3_	O_g2_-α(O_u1_+O_u2_+O_u3_)	I_e1_+O_g3_+I_e3_-β(O_e1_+O_e3_)
(Incentives, negative implementation, negative enforcement)	I_g3_-O_g1_-O_g2_	O_g2_-α(O_u1_+O_u2_)	I_e1_-β(O_e1_+O_e3_)-I_g3_
(No incentives, active implementation, active enforcement)	I_g1_+I_g2_-O_g1_	I_u3_-α(O_u1_+O_u2_+O_u3_)	I_e3_-β(O_e1_+O_e3_)
(No incentives, positive implementation, negative execution)	I_g1_+I_g3_-O_g1_	I_u3_-α(O_u1_+O_u2_)	-β(O_e1_+O_e3_)-I_e3_
(No incentives, negative implementation, positive implementation)	I_g2_-O_g1_	0	I_e3_-β(O_e1_+O_e3_)
(No incentives, negative implementation, negative enforcement)	I_g3_-O_g1_	-α(O_u1_+O_u2_)	-β(O_e1_+O_e3_)-I_e3_

## 3 Analysis of evolutionary games

### 3.1 Replicative dynamic modeling of game subjects

Since GM, US and EP are rational game subjects, based on replication dynamics and evolutionary game theory, this paper analyzes the dynamic adjustment of game subjects and their strategies, constructs the replication dynamics model of each game subject, and analyzes the evolutionary stability strategy [54, 55].

Let the expected return on GM’s choice of the "incentive" strategy be U_11_, then the expected return on the "disincentive" strategy be U_12_, and the average expected return be U_1_, as follows:

$$\begin{array}{ll} U_{11} & =yz\left(I_{g1}+I_{g2}-O_{g1}-O_{g2}-O_{g3}-O_{g4}\right)\\ & +y\left(1-z\right)\left(I_{g1}+I_{g3}-O_{g1}-O_{g2}-O_{g4}\right)\\ & +\left(1-y\right)z\left(I_{g2}-O_{g1}-O_{g2}-O_{g3}\right)\\ & +\left(1-y\right)\left(1-z\right)(I_{g3}-O_{g1}-O_{g2}) \end{array}$$

$$\begin{array}{ll} U_{12} & =yz\left(I_{g1}+I_{g2}-O_{g1}\right)+y\left(1-z\right)\left(I_{g1}+I_{g3}-O_{g1}\right)\\ & +\left(1-y\right)z\left(I_{g2}-O_{g1}\right)+\left(1-y\right)\left(1-z\right)(I_{g3}-O_{g1}) \end{array}$$

$$U_{1}=xU_{11}+(1-x)U_{12}$$

Therefore, the equation for the replication dynamics of the "incentive" strategy adopted by GM is:

$$\begin{array}{ll} F\left(x\right) & =\frac{dx}{dt}=x\left(U_{11}-U_{1}\right)\\ & =x\left(1-x\right)\left(U_{11}-U_{12}\right)\\ & =x\left(1-x\right)\left[2y\left(O_{g2}-zO_{g2}-zO_{g4}\right)+yO_{g4}-zO_{g3}-O_{g2}\right] \end{array}$$

Similarly, let the expected return of US choosing the "active implementation" strategy be U_21_, the expected return of US choosing the "passive implementation" strategy be U_22_, and the average expected return be U_2_, as follows:

$$\begin{array}{ll} U_{21} & =xz\left(O_{g2}+O_{g4}+I_{u3}-\alpha \left(O_{u1}+O_{u2}+O_{u3}\right)\right)\\ & +x\left(1-z\right)\left(O_{g2}+O_{g4}+I_{u3}-\alpha \left(O_{u1}+O_{u2}+O_{u3}\right)\right)\\ & +\left(1-x\right)z\left(I_{u3}-\alpha \left(O_{u1}+O_{u2}+O_{u3}\right)\right)\\ & +\left(1-x\right)\left(1-z\right)(I_{u3}-\alpha (O_{u1}+O_{u2}+O_{u3})) \end{array}$$

$$\begin{array}{ll} U_{22} & =xz\left(O_{g2}-\alpha \left(O_{u1}+O_{u2}+O_{u3}\right)\right)\\ & +x\left(1-z\right)\left(O_{g2}-\alpha \left(O_{u1}+O_{u2}+O_{u3}\right)\right)\\ & +\left(1-x\right)z\left(-\alpha \left(O_{u1}+O_{u2}+O_{u3}\right)\right)\\ & +\left(1-x\right)\left(1-z\right)\left(-\alpha \left(O_{u1}+O_{u2}+O_{u3}\right)\right) \end{array}$$

$$U_{2}=yU_{21}+\left(1-y\right)U_{22}$$

Therefore, the dynamic equation of replication for US adopting the "active implementation" strategy is:

$$\begin{array}{ll} F\left(y\right) & =\frac{dy}{dt}=y\left(U_{21}-U_{2}\right)\\ & =y\left(1-y\right)\left(U_{21}-U_{22}\right)\\ & =y\left(1-y\right)\left(xO_{g4}+O_{g2}+\left(z-xz\right)\left(-\alpha \left(O_{u1}+O_{u2}+O_{u3}\right)\right)\right) \end{array}$$

Similarly, let the expected return of EP choosing the "active execution" strategy be U_31_, the expected return of EP choosing the "passive execution" strategy be U_32_, and the average expected return be U_3_, as follows:

$$\begin{array}{ll} U_{31} & =xy\left(I_{e1}+O_{g3}+I_{e3}-\beta \left(O_{e1}+O_{e3}\right)\right)\\ & +x\left(1-y\right)\left(I_{e1}+O_{g3}+I_{e3}-\beta \left(O_{e1}+O_{e3}\right)\right)\\ & +\left(1-x\right)y\left(I_{e1}+O_{g3}+I_{e3}-\beta \left(O_{e1}+O_{e3}\right)\right)\\ & +\left(1-x\right)\left(1-y\right)\left(I_{e1}+O_{g3}+I_{e3}-\beta \left(O_{e1}+O_{e3}\right)\right) \end{array}$$

$$\begin{array}{ll} U_{32} & =xy\left(I_{e1}-\beta \left(O_{e1}+O_{e3}\right)-I_{g3}\right)\\ & +x\left(1-y\right)\left(I_{e1}-\beta \left(O_{e1}+O_{e3}\right)-I_{g3}\right)\\ & +\left(1-x\right)y\left(I_{e1}-\beta \left(O_{e1}+O_{e3}\right)-I_{g3}\right)\\ & +\left(1-x\right)\left(1-y\right)\left(I_{e1}-\beta \left(O_{e1}+O_{e3}\right)-I_{g3}\right) \end{array}$$

$$U_{3}=zU_{31}+\left(1-z\right)U_{32}$$

Therefore, the replication dynamics equation for EP’s adopting the "aggressive enforcement" strategy is:

$$\begin{array}{ll} F\left(z\right) & =\frac{dz}{dt}=z\left(U_{31}-U_{3}\right)\\ & =z\left(1-z\right)\left(U_{31}-U_{32}\right)\\ & =z\left(1-z\right)(xyO_{g3}-yO_{g3}+I_{e1}) \end{array}$$

### 3.2 GM evolutionary stabilization

A partial derivation of the equations for the replication dynamics of the GM’s choice of "incentive" strategy is obtained:

$$\frac{dF(x)}{dx}=(1-2x)[2y\left(O_{g2}-zO_{g2}-zO_{g4}\right)+yO_{g4}-zO_{g3}-O_{g2}]$$

**Fig 3:**
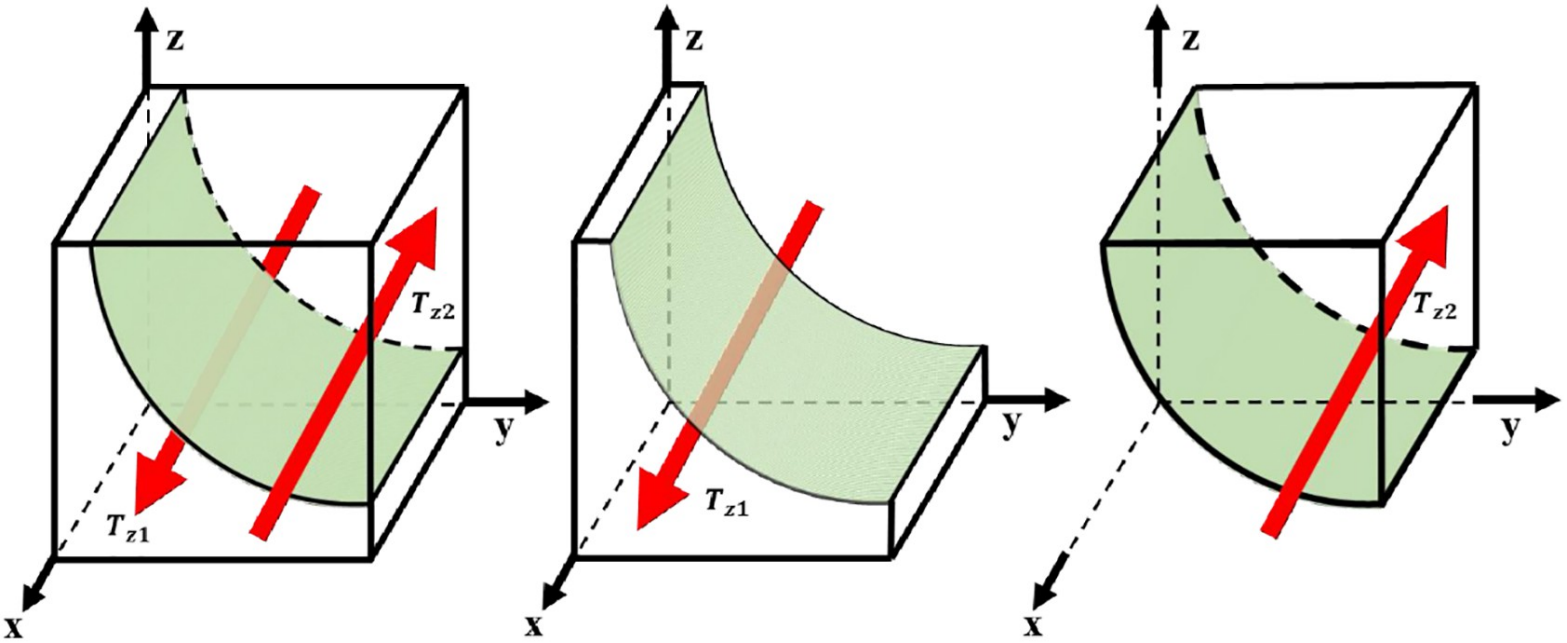
GM evolutionary stabilization strategy.

According to the stability theorem of differential equation, the probability of GM choosing "incentive" to be in steady state must satisfy [56]: *F*(*x*) = 0 and $\frac{dF(x)}{dx}<0$. When $z=\frac{2yO_{g2}+yO_{g4}-O_{g2}}{2yO_{g2}+2yO_{g4}+O_{g3}}$, then any level is in the steady state. stable state; when $z>\frac{2yO_{g2}+yO_{g4}-O_{g2}}{2yO_{g2}+2yO_{g4}+O_{g3}}$, then *y* = 0 is an evolutionary stable strategy; when $z<\frac{2yO_{g2}+yO_{g4}-O_{g2}}{2yO_{g2}+2yO_{g4}+O_{g3}}$, then *y* = 1 is an evolutionary stable strategy. As a result, the GM chooses the "incentive" replication dynamics and the trend of evolutionary stabilization strategy is shown in Fig 3.

The volume of region T_*z*1_ represents the probability that the GM is "motivated" and the volume of region T_*z*2_ represents the probability that the GM is "not motivated".


$$T_{z1}=\iint \frac{2yO_{g2}+yO_{g4}-O_{g2}}{2yO_{g2}+2yO_{g4}+O_{g3}}dxdy$$

$$T_{z2}=1-\iint \frac{2yO_{g2}+yO_{g4}-O_{g2}}{2yO_{g2}+2yO_{g4}+O_{g3}}dxdy$$

Proposition 1: The probability of GM choosing "incentive" will decrease with the increase of the probability of US "active implementation" and EP "active implementation".

Proof 1: From the partial derivation of the replicated dynamic model of the GM’s choice of "incentives", we obtain the correlation function between the probability *x* that the GM regulates and the probability z that the EP chooses to "actively enforce":

$$x\left\{\begin{array}{cc} 0 & z>\frac{2yO_{g2}+yO_{g4}-O_{g2}}{2yO_{g2}+2yO_{g4}+O_{g3}}\;\\ \left[0,1\right] & z=\frac{2yO_{g2}+yO_{g4}-O_{g2}}{2yO_{g2}+2yO_{g4}+O_{g3}}\\ 1 & z<\frac{2yO_{g2}+yO_{g4}-O_{g2}}{2yO_{g2}+2yO_{g4}+O_{g3}} \end{array}\right.$$

When $z=\frac{2yO_{g2}+yO_{g4}-O_{g2}}{2yO_{g2}+2yO_{g4}+O_{g3}}$, then any level is in steady state. When $z>\frac{2yO_{g2}+yO_{g4}-O_{g2}}{2yO_{g2}+2yO_{g4}+O_{g3}}$, *x* = 0 is an evolutionarily stable strategy, and it can be concluded that when the probability of the EP being "actively implemented" is higher than a certain value, the GM prefers not to incentivize, saving financial investment, and the chosen strategy stabilizes at 0. When $z<\frac{2yO_{g2}+yO_{g4}-O_{g2}}{2yO_{g2}+2yO_{g4}+O_{g3}}$, *x* = 1 is the evolutionary stable strategy, which can be concluded that when the probability of EP "active implementation" is lower than a certain value, the GM will increase the support to increase the incentives given to the EP, and the chosen strategy is stable at 1. is stabilized at 1.

Similarly, the correlation function between the probability x of GM "motivation" and the probability y of US "active implementation" can be derived. When $y>\frac{zO_{g3}+O_{g2}}{2O_{g2}+2zO_{g2}-2zO_{g4}+O_{g4}}$, *x* = 0 is an evolutionarily stable strategy, and the GM decreases incentives and prefers not to incentivize when the US is biased in favor of "active implementation". Therefore, the strategy chosen by the GM is stabilized at 0. When $y<\frac{zO_{g3}+O_{g2}}{2O_{g2}+2zO_{g2}-2zO_{g4}+O_{g4}}$, *x* = 1 is an evolutionarily stable strategy, when the US tends to be "negatively implemented", the GM will increase the level of support, and the strategy is stabilized at 1.

Proposition 2: The probability that a GM chooses "incentives" decreases as the cost of financial support (*O*_*g*2_), the cost of compensating EP’s (*O*_*g*3_), and the incentive for improving the overall strength of the US (*O*_*g*4_) increase.

Proof 2: A partial derivation of the financial cost of schooling (*O*_*g*2_), the cost of compensating EP’s (*O*_*g*3_), and the incentive to improve the overall strength of the US (*O*_*g*4_) that affects the effect of GM "incentivization" yields: $\frac{\partial \left(T_{z1}\right)}{\partial O_{g2}}<0$, which suggests that an increase in the financial support for schooling leads to a decrease in the probability of the GM’s supervising the school. A decrease in the probability. $\frac{\partial \left(T_{z1}\right)}{\partial O_{g3}}<0$, indicating that an increase in the cost of compensation to EP’s leads to a decrease in the probability of GM regulation. $\frac{\partial \left(T_{z1}\right)}{\partial O_{g4}}<0$. It shows that the increase of GM’s additional reward for the improvement of US comprehensive strength will reduce the probability of GM supervision.

### 3.3 US evolutionary gaming strategy

A partial derivation of the equation for the dynamics of replication for US choosing the "active implementation" strategy yields:

$$\frac{dF(y)}{dy}=\left(1-2y\right)(xO_{g4}+O_{g2}+\left(z-xz\right)(-\alpha (O_{u1}+O_{u2}+O_{u3})))$$

**Fig 4:**
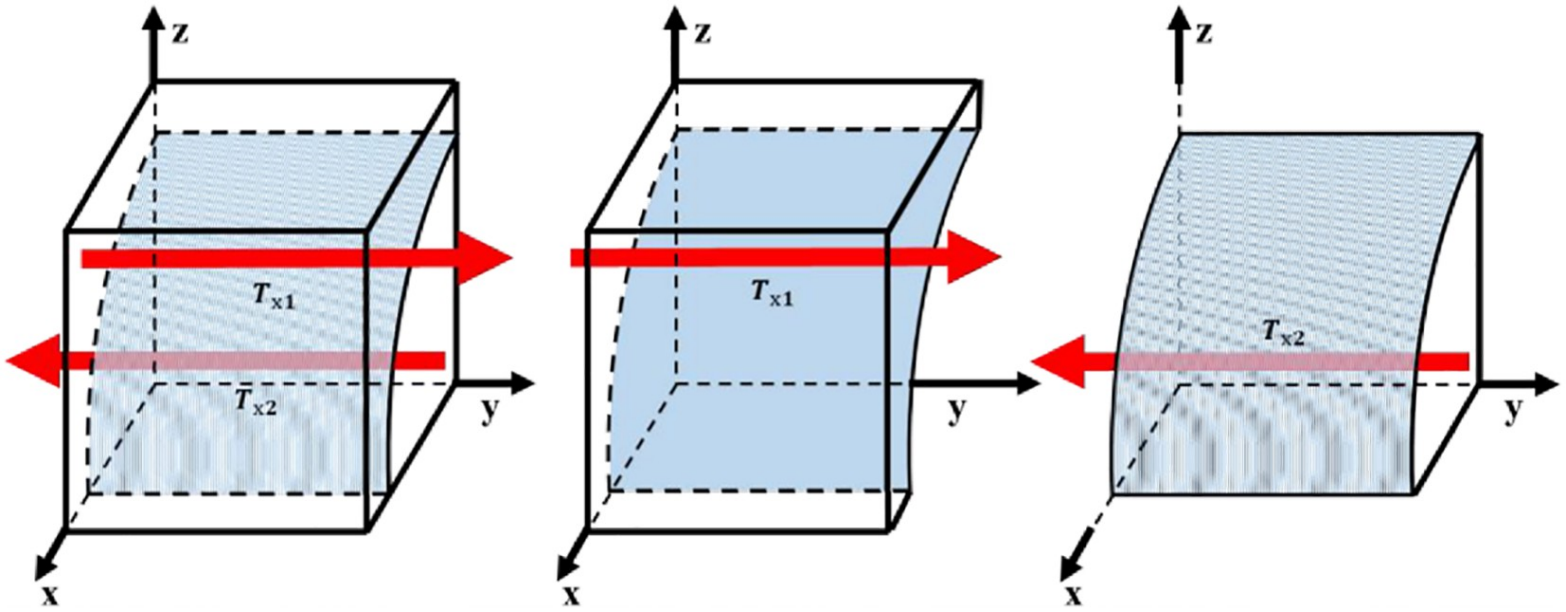
US evolutionary stabilization strategy.

When $x=\frac{z(\alpha \left(O_{u1}+O_{u2}+O_{u3}\right)-O_{g2})}{O_{g4}+Z(\alpha \left(O_{u1}+O_{u2}+O_{u3}\right))}$, then any level is in the steady state; when $x<\frac{z(\alpha \left(O_{u1}+O_{u2}+O_{u3}\right)-O_{g2})}{O_{g4}+Z(\alpha \left(O_{u1}+O_{u2}+O_{u3}\right))}$ and *y* = 1 is the evolutionary stabilization strategy; when $x>\frac{z(\alpha \left(O_{u1}+O_{u2}+O_{u3}\right)-O_{g2})}{O_{g4}+Z(\alpha \left(O_{u1}+O_{u2}+O_{u3}\right))}$, then *y* = 0 is the evolutionary stabilization strategy. Thus, the trend of replication dynamics and evolutionary stabilization strategy of US choosing "active implementation" is shown in Fig 4.

The volume of region T_*x*1_ represents the probability that the US is "motivated" and the volume of region T_*x*2_ represents the probability that the US is "not motivated".


$$T_{x1}=\iint \frac{z(\alpha \left(O_{u1}+O_{u2}+O_{u3}\right)-O_{g2})}{O_{g4}+z(\alpha \left(O_{u1}+O_{u2}+O_{u3}\right))}dydz$$

$$T_{x2}=1-\iint \frac{z(\alpha \left(O_{u1}+O_{u2}+O_{u3}\right)-O_{g2})}{O_{g4}+z(\alpha \left(O_{u1}+O_{u2}+O_{u3}\right))}dydz$$

Proposition 3: The probability that US chooses "active implementation" increases with the probability that GM "incentivizes" and EP "active implementation".

Proof 3: By taking the derivative from the replication dynamic model in which US chooses "active implementation", the correlation function between the probability y of US choosing "active implementation" and the probability x of GM choosing "incentive" can be obtained:

$$y\left\{\begin{array}{cc} 0 & x<\frac{z(\alpha \left(O_{u1}+O_{u2}+O_{u3}\right)-O_{g2})}{O_{g4}+z(\alpha \left(O_{u1}+O_{u2}+O_{u3}\right))}\;\\ \left[0,1\right] & x=\frac{z(\alpha \left(O_{u1}+O_{u2}+O_{u3}\right)-O_{g2})}{O_{g4}+Z(\alpha \left(O_{u1}+O_{u2}+O_{u3}\right))}\\ 1 & x>\frac{z(\alpha \left(O_{u1}+O_{u2}+O_{u3}\right)-O_{g2})}{O_{g4}+z(\alpha \left(O_{u1}+O_{u2}+O_{u3}\right))} \end{array}\right.$$

When $x>\frac{z(\alpha \left(O_{u1}+O_{u2}+O_{u3}\right)-O_{g2})}{O_{g4}+z(\alpha \left(O_{u1}+O_{u2}+O_{u3}\right))}$, *y* = 1 is an evolutionarily stable strategy, and it can be concluded that when the probability of the GM being "incentivized" is higher than a certain value, the US prefers "active implementation", and the chosen strategy is stable at 1. "active implementation" and the chosen strategy stabilizes at 1. When $x<\frac{z(\alpha \left(O_{u1}+O_{u2}+O_{u3}\right)-O_{g2})}{O_{g4}+z(\alpha \left(O_{u1}+O_{u2}+O_{u3}\right))}$, *y* = 0 is an evolutionarily stable strategy, which leads to the conclusion that US prefers "active implementation" when the probability of GM "incentive" is higher than a certain value, and it can be concluded that US prefers to implement "active implementation" when the probability of GM "incentive" is higher than a certain value. "incentive" probability is lower than a certain value, US will tend to save costs and reduce the investment in professional construction, faculty investment and integration with EP, and its choice of strategy is stabilized at 0.

Similarly, the correlation function between the probability y of US "active implementation" and the probability z of EP "active execution" can be derived. When $z>\frac{xO_{g4}+O_{g2}}{\alpha \left(1-x\right)\left(O_{u1}+O_{u2}+O_{u3}\right)}$, *y* = 1 is an evolutionarily stable strategy, and when the probability of EP’s "active implementation" is higher than a certain value, the US tends to "actively implement". When the probability of EP’s "positive implementation" is higher than a certain value, US tends to "positively implement", and is more inclined to positively cooperate with EP, so the strategy chosen by US is stabilized at 1. When $z<\frac{xO_{g4}+O_{g2}}{\alpha \left(1-x\right)\left(O_{u1}+O_{u2}+O_{u3}\right)}$, *y* = 0 is an evolutionarily stable strategy, and it can be concluded that when the probability of EP’s "positive implementation" is lower than a certain value, US tends to "negatively implement", and is more inclined to "positively implement". When the probability of EP "negative implementation" is lower than a certain value, US tends to "negative implementation", US will reduce the intensity of school-enterprise cooperation, and its strategy is stabilized at 0.

Proposition 4: The probability of US selection increases with financial support and additional incentives, and decreases with the cost of professional development, faculty inputs, and inputs and payment coefficients with EP.

Proof 4: The cost of financial support (*O*_*g*2_), additional incentives received (*O*_*g*4_), investment in professional development (*O*_*u*1_), faculty investment (*O*_*u*2_), investment in cooperation with EP’s (*O*_*u*3_), and the coefficient of disbursement (*α*) affecting the effect of the US’s "active implementation" can be obtained by taking the partial derivatives: $\frac{\partial \left(T_{x1}\right)}{\partial O_{g2}}>0,\;\frac{\partial \left(T_{x1}\right)}{\partial O_{g4}}>0$, this suggests that an increase in financial support and additional incentives received by the US leads to an increase in the probability that the US is "actively implementing". $\frac{\partial \left(T_{x1}\right)}{\partial O_{u1}}<0,\;\frac{\partial \left(T_{x1}\right)}{\partial O_{u2}}<0,\;\frac{\partial \left(T_{x1}\right)}{\partial O_{u3}}<0,\;\frac{\partial \left(T_{x1}\right)}{\partial \alpha }<0$, which indicates that an increase in the cost of professional development, investment in faculty, and investment in the cooperation with EPs, and the coefficient of payment leads to a decrease in the probability of "active implementation" of US.

### 3.4 EP evolutionary game strategy

A partial derivation of the equation for the replication dynamics of the EP choosing the "aggressive enforcement" strategy is obtained:

$$\frac{dF(z)}{dz}=\left(1-2z\right)(xyO_{g3}-yO_{g3}+I_{e1})$$

**Fig 5:**
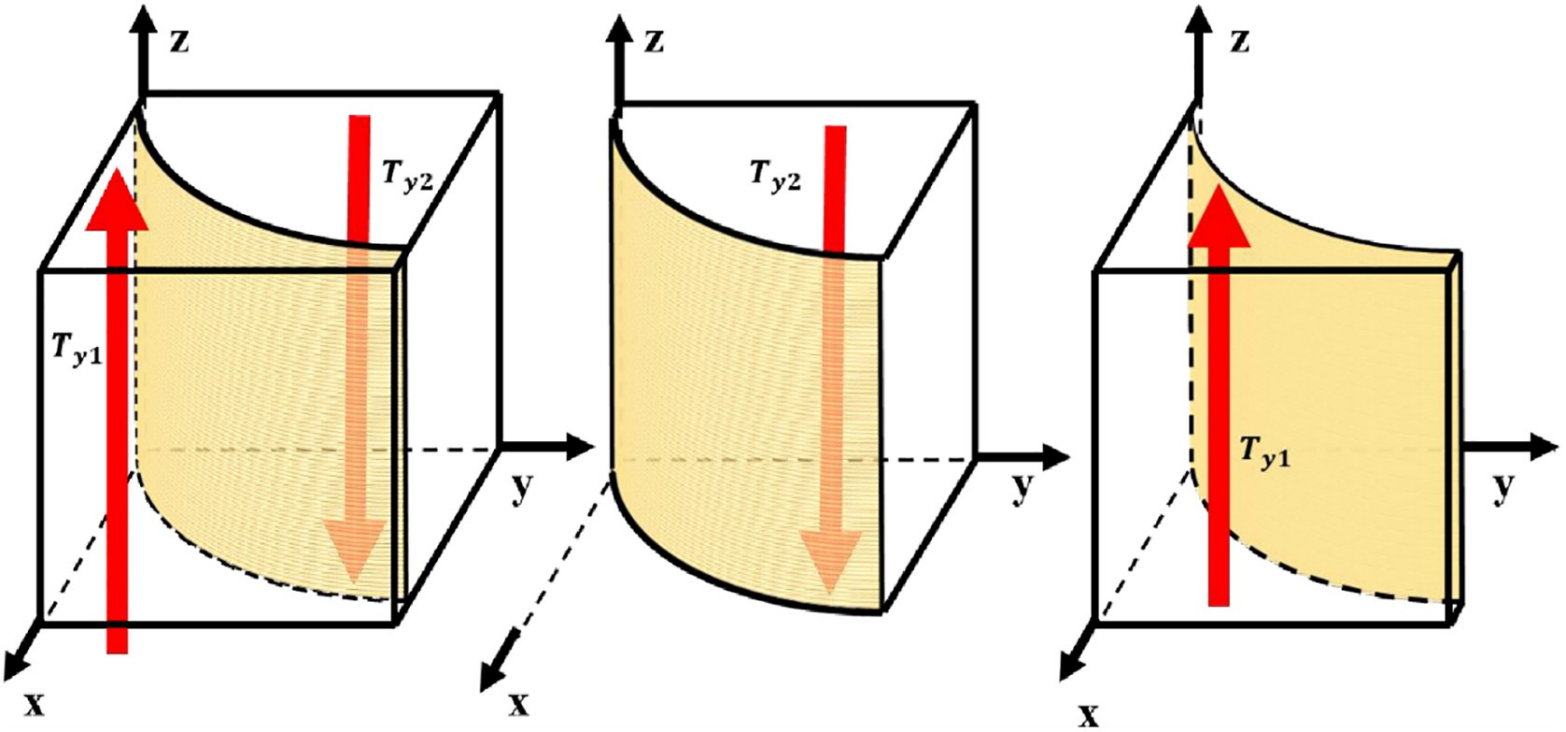
EP evolution stabilization strategy.

When $y=\frac{O_{g3}-I_{e1}}{xO_{g3}}$, any level is stable; when $y<\frac{yO_{g3}-I_{e1}}{xO_{g3}}$, z = 1 is the evolutionary stable strategy; when $y>\frac{O_{g3}-I_{e1}}{xO_{g3}}$, z = 0 is the evolutionary stable strategy. As a result, EP chooses the replication dynamics of "active execution" and the trend of evolutionary stabilization strategy is shown in Fig 5.

The volume of region *T*_*y*1_ represents the probability of EP "positive implementation" and the volume of region *T*_*y*2_ represents the probability of EP "negative implementation".


$$T_{y1}=\iint \frac{O_{g3}-I_{e1}}{xO_{g3}}dxdz$$

$$T_{y2}=1-\iint \frac{O_{g3}-I_{e1}}{xO_{g3}}dxdz$$

Proposition 5: The probability that an EP chooses "active implementation" decreases as the probabilities of GM "motivation" and US "active implementation" increase.

Proof 5: By partial derivation of the replicated dynamic model of EP’s choice of "active implementation", the probability z of EP’s choice of "active implementation" can be obtained as a function of the probability y of US’s choice of "active implementation":

$$z\left\{\begin{array}{cc} 0 & y<\frac{O_{g3}-I_{e1}}{xO_{g3}}\;\\ \left[0,1\right] & y=\frac{O_{g3}-I_{e1}}{xO_{g3}}\\ 1 & y>\frac{O_{g3}-I_{e1}}{xO_{g3}} \end{array}\right.$$

When $y<\frac{O_{g3}-I_{e1}}{xO_{g3}}$, *z* = 1 is the evolutionary stabilization strategy, and it can be concluded that when the probability of US "active implementation" is lower than a certain value, the EP prefers "active implementation", and the chosen strategy is stabilized at 1. When $y>\frac{O_{g3}-I_{e1}}{xO_{g3}}$, *z* = 0 is the evolutionary stabilization strategy, and it can be concluded that when the probability of US "active implementation" is higher than a certain value, the EP prefers "passive implementation", and reduces the inputs of the EP’s fusion, and its chosen strategy is stabilized at 0.

When $x<\frac{O_{g3}-I_{e1}}{yO_{g3}}$, *z* = 1 is the evolutionary stable strategy, which can be concluded that when the probability of GM’s "incentive" is lower than a certain value, EP prefers "positive execution", and the chosen strategy is stabilized at 1. When $x>\frac{O_{g3}-I_{e1}}{yO_{g3}}$, *z* = 0 is the evolutionary stable strategy, which can be concluded that when the probability of GM’s "incentive" is higher than a certain value, EP prefers "negative execution", and its chosen strategy is stabilized at 0.

Proposition 6: The probability that an EP chooses to "actively implement" increases with GM incentives and policy support from the GM.

Proof 6: For the GM reward (*O*_*g*3_) and the policy support from GM (*I*_*e*1_) that affects the effect of EP’s "active implementation", the partial derivation can be obtained as follows: $\frac{\partial \left(T_{z1}\right)}{\partial O_{g3}}>0,\;\frac{\partial \left(T_{z1}\right)}{\partial I_{e1}}>0$, which indicates that the probability of EP choosing to "implement aggressively" increases with the increase of the GM reward and the policy support from GM.

### 3.5 Stability analysis of the evolution of the strategies of the three main parties

For between GM, US and EP, this paper only explores the asymptotic stability of the eight points *E*_1_(0,0,0), *E*_2_(0,0,1), *E*_3_(0,1,0), *E*_4_(0,1,1), *E*_5_(1,0,0), *E*_6_(1,0,1), *E*_7_(1,1,0) and *E*_8_(1,1,1). The stability analysis is determined with the help of Jacobi matrix which is given below [57]:

$$\left[\begin{array}{ccc} \left(1-2x\right)\left[2y\left(O_{g2}-zO_{g2}-zO_{g4}\right)+yO_{g4}-zO_{g3}-O_{g2}\right] & x\left(1-x\right)\left[2\left(O_{g2}-zO_{g2}-zO_{g4}\right)+O_{g4}\right] & x\left(1-x\right)\left[2y\left(-O_{g2}-O_{g4}\right)-O_{g3}\right]\\ y\left(1-y\right)\left[O_{g4}+z\alpha \left(O_{u1}+O_{u2}+O_{u3}\right)\right] & \left(1-2y\right)(xO_{g4}+O_{g2}+(xz-z)\alpha (O_{u1}+O_{u2}+O_{u3}) & y\left(1-y\right)(x-1)\alpha (O_{u1}+O_{u2}+O_{u3})\\ z\left(1-z\right)yO_{g3} & z\left(1-z\right)\left(xO_{g3}-O_{g3}\right) & \left(1-2z\right)\left(xyO_{g3}-yO_{g3}+I_{e1}\right) \end{array}\right]$$

According to the Liaplov stability theory [58], the asymptotic stability properties of the system at the equilibrium point are judged by analyzing the eigenvalues of the system Jacobi matrix, i.e., a sufficient condition for the asymptotic stability of the system is that all the eigenvalues of the Jacobi matrix are less than 0. Table 3 summarizes the stability determinations at each equilibrium point.

**Table 3:** Stability judgment of each equilibrium point.

Balance points	*λ*_1_	*λ*_2_	*λ*_3_	Stability
*E*_1_(0,0,0)	*I*_*e*1_ (+)	*O*_*g*2_ (+)	−*O*_*g*2_ (−)	Unstable
*E*_2_(0,0,1)	−*I*_*e*1_ (−)	−*O*_*g*2_ − *O*_*g*3_ (−)	*O*_*g*2_ − *α*(*O*_*u*1_ + *O*_*u*2_ + *O*_*u*3_) (*s*)	satisfying (a) is ESS
*E*_3_(0,1,0)	*O*_*g*2_ + *O*_*g*4_ (+)	*I*_*e*1_ − *O*_*g*3_ (*s*)	−*O*_*g*2_ (−)	Unstable
*E*_4_(0,1,1)	*O*_*g*3_ − *I*_*e*1_ (*s*)	−*O*_*g*2_ − *O*_*g*3_ − *O*_*g*4_ (−)	*α*(*O*_*u*1_ + *O*_*u*2_ + *O*_*u*3_) − *O*_*g*2_ (*s*)	satisfying (b) is ESS
*E*_5_(1,0,0)	*I*_*e*1_ (+)	*O*_*g*2_ (+)	*O*_*g*2_ + *O*_*g*4_ (+)	Unstable
*E*_6_(1,0,1)	*O*_*g*2_ + *O*_*g*3_ (+)	*O*_*g*2_ + *O*_*g*4_ (+)	−*I*_*e*1_ (−)	Unstable
*E*_7_(1,1,0)	*I*_*e*1_ (+)	−*O*_*g*2_ − *O*_*g*4_ (−)	−*O*_*g*2_ − *O*_*g*4_ (−)	Unstable
*E*_8_(1,1,1)	−*I*_*e*1_ (−)	−*O*_*g*2_ − *O*_*g*4_ (−)	*O*_*g*2_ + *O*_*g*3_ + *O*_*g*4_ (+)	Unstable

Notes in the table denotes sign uncertainty; ESS denotes stable strategy; if condition (a and b) is not satisfied, then it is an unstable point. Condition (a): *O*_*g*2_ − *α*(*O*_*u*1_ + *O*_*u*2_ + *O*_*u*3_) < 0. Condition (b): *O*_*g*3_ − *I*_*e*1_ < 0 and *α*(*O*_*u*1_ + *O*_*u*2_ + *O*_*u*3_) − *O*_*g*2_ < 0.

As can be seen from Table 3, when satisfying *O*_*g*2_ − *α*(*O*_*u*1_ + *O*_*u*2_ + *O*_*u*3_) < 0, *E*_2_(0,0,1) is the stabilization point (ESS) of the replicated dynamical system, and it can be seen that the financial support given to the US by GM should be less than the total investment of the US in running the school by *O*_*g*2_ − *α*(*O*_*u*1_ + *O*_*u*2_ + *O*_*u*3_) < 0. When satisfy *O*_*g*3_ − *I*_*e*1_ < 0 and *α*(*O*_*u*1_ + *O*_*u*2_ + *O*_*u*3_) − *O*_*g*2_ < 0, *E*_4_(0,1,1) is ESS, from *O*_*g*3_ − *I*_*e*1_ < 0, it is known that from the EP point of view, the reward given by GM to EP should be smaller than the policy support given by GM, and the policy reward given by GM is more conducive to the stability of the system; from *α*(*O*_*u*1_ + *O*_*u*2_ + *O*_*u*3_) − *O*_*g*2_ < 0 it is known that in terms of US, US prefers to reduce the cost of schooling.

## 4 Evolutionary simulation analysis

**Fig 6:**
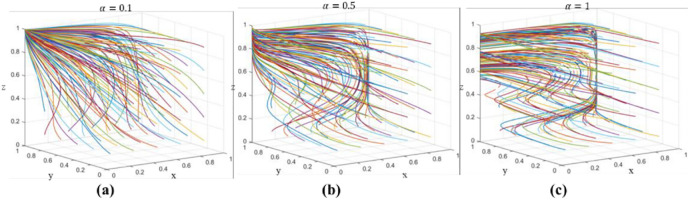
Evolutionary simulation analysis results.

The following numerical simulation has been carried out by MATLAB 2018, which can visualize the dynamic evolution process of the stakeholders to determine the optimal path of ETPP. In the stability analysis of the model, both *E*_2_(0,0,1) and *E*_4_(0,1,1) may be the ESS of the model, i.e., the strategy combinations of GM, US, and EP (no incentives, negative implementation, and positive implementation) and (no incentives, positive implementation, and positive implementation) are the two evolutionary stable strategy combinations. With α = 0.1, 0.5 and 1, 3D simulation analysis of the evolution of different initial strategies is performed under the condition of guaranteeing Table 3(b), and the simulation results are shown in Fig 6. The 3D dynamic evolution simulation shows that, independent of the initial strategies of the three parties, the final convergence is to *E*_4_(0,1,1), that is, GM chooses "no incentive" and US chooses "active implementation". ", US chooses "active implementation", and GM chooses "active execution". Therefore, the above theoretical analysis has been verified. Therefore, GM believes that policy support for EP should be strengthened to increase the possibility of active implementation in the United States. It can be seen that the simulation analysis is consistent with the conclusion of all parties’ strategy stability analysis, which is effective and has practical significance for ETPP.

**Fig 7:**
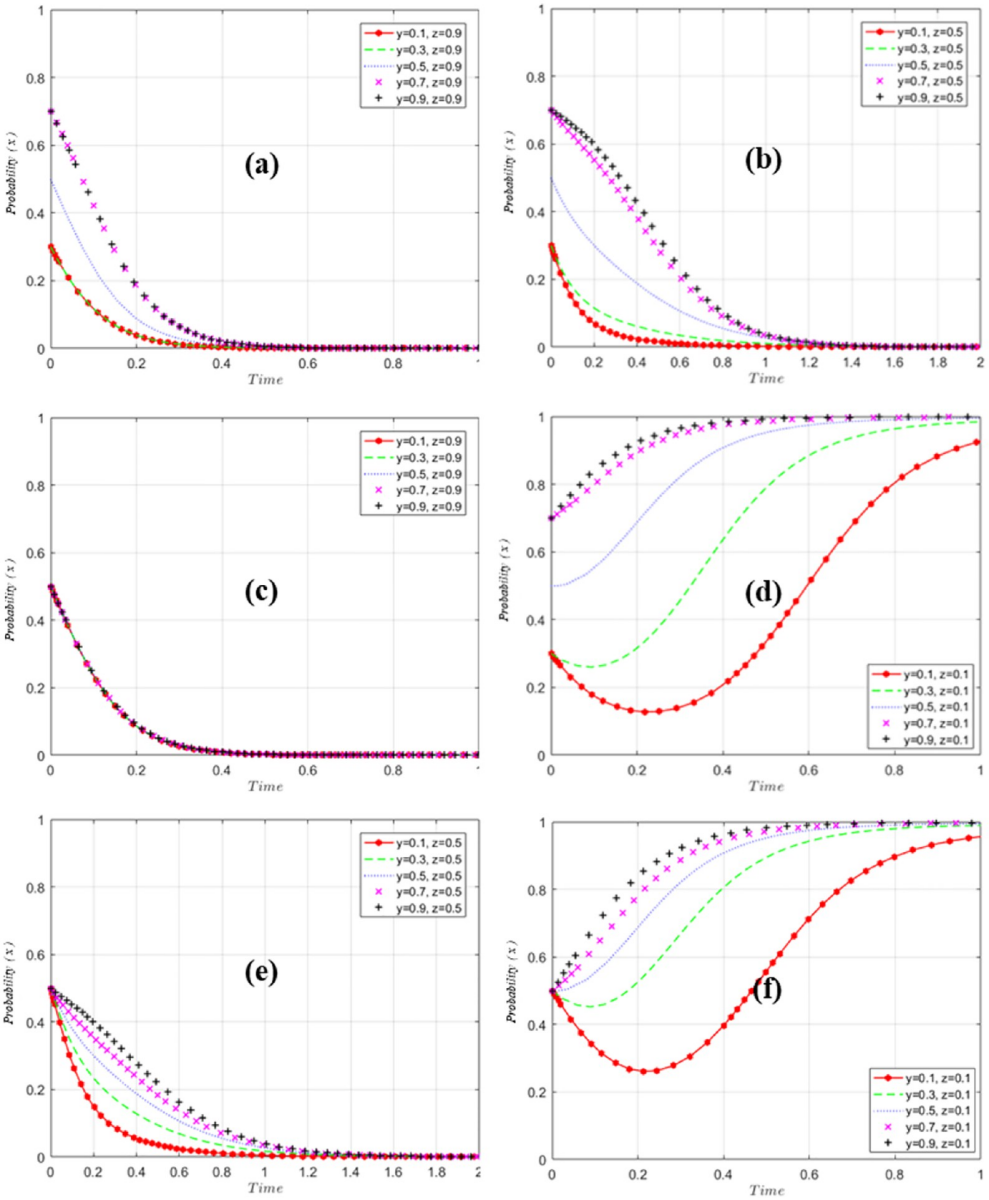
GM excitation evolution simulation analysis results.

Further, the evolution of GM, US and EP is simulated and analyzed by assigning values to each parameter and using Matlab tool. In this paper, the variables are assigned values such that *O*_*g*2_ = 50, *O*_*g*3_ = 10, *O*_*g*4_ = 30, *O*_*u*1_ = 50, *O*_*u*2_ = 20, *O*_*u*3_ = 20, *I*_*e*1_ = 20, The parameter setting is based on the research methods of Gao and Sun et al. [59, 60], and the simulation results are shown in Fig 7.

Fig 7 simulation analysis results of GM excitation evolution. When EP shows positive and positive matching and US pursues EPTT, regardless of the probability of GM’s initial selection of incentives, GM will eventually stabilize at probability 0 (Fig 7a). When EP is negative, the probability of GM finally stabilizing at 1 (Fig 7d and 7f). Further analysis, EP, as an irreplaceable subject in EPTT, shows that under the condition of controlling EP to maintain a high probability, by adjusting the strategy of US, the probability of GM is mainly related to the probability of EP’s positive implementation (Fig 7c), and is not much affected by the probability of US. Further lowering the probability of EP to z = 0.5 (Fig 7b) eventually stabilizes at a probability of 0 regardless of the initial probability of the GM’s choice of regulation, but stabilizes significantly slower than z = 0.9 (Fig 7a), and when tuning down the probability of EP the probability of GM incentives rapidly converges to 0 as the probability of US implementation increases (Fig 7e). It indicates that EP occupies a more important position in influencing GM decision-making, and the more positively US is implemented, the faster the GM probability tends to 0. EP’s behavioral decision affects the speed of GM’s decision-making or the strength of implementation of related policies. When US is implemented negatively and EP is implemented negatively to maximize self-interest (Fig 7d), the probability of GM choosing "incentives" stabilizes at 1. US is very important in this evolution process, and its active implementation has accelerated the evolution process (Fig 7f).

**Fig 8:**
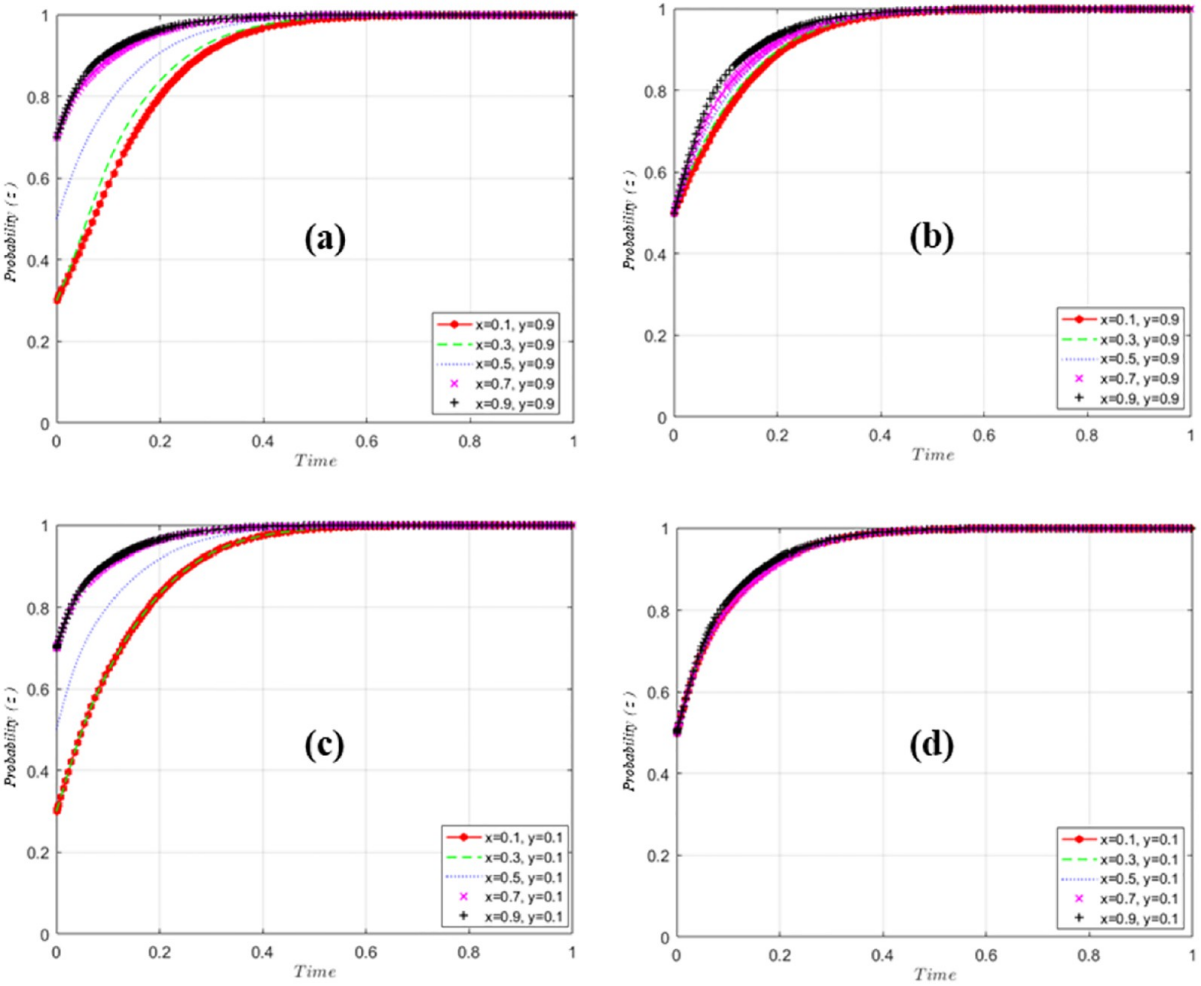
Results of US implementation evolution simulation analysis.

As shown in Fig 8a, when GM is motivated, regardless of the probability that US will take the initiative at first, US will tend to take the initiative to improve the strength of US education and the overall ranking of US, and the final probability will stabilize at 1; at this time, regardless of the adjustment of the probability of the EP implementation, the impact on the strategy of the US is small (Fig 8b). When GM chooses not to incentivize, adjusting the probability of EP, it is found that the final probability of US stabilizes at 1 (Fig 8c), and at this time, adjusting the initial probability of US has a smaller impact on the speed of convergence of US’s strategy (Fig 8d). This shows that the probability changes of GM and EP have little impact on American strategy.

**Fig 9:**
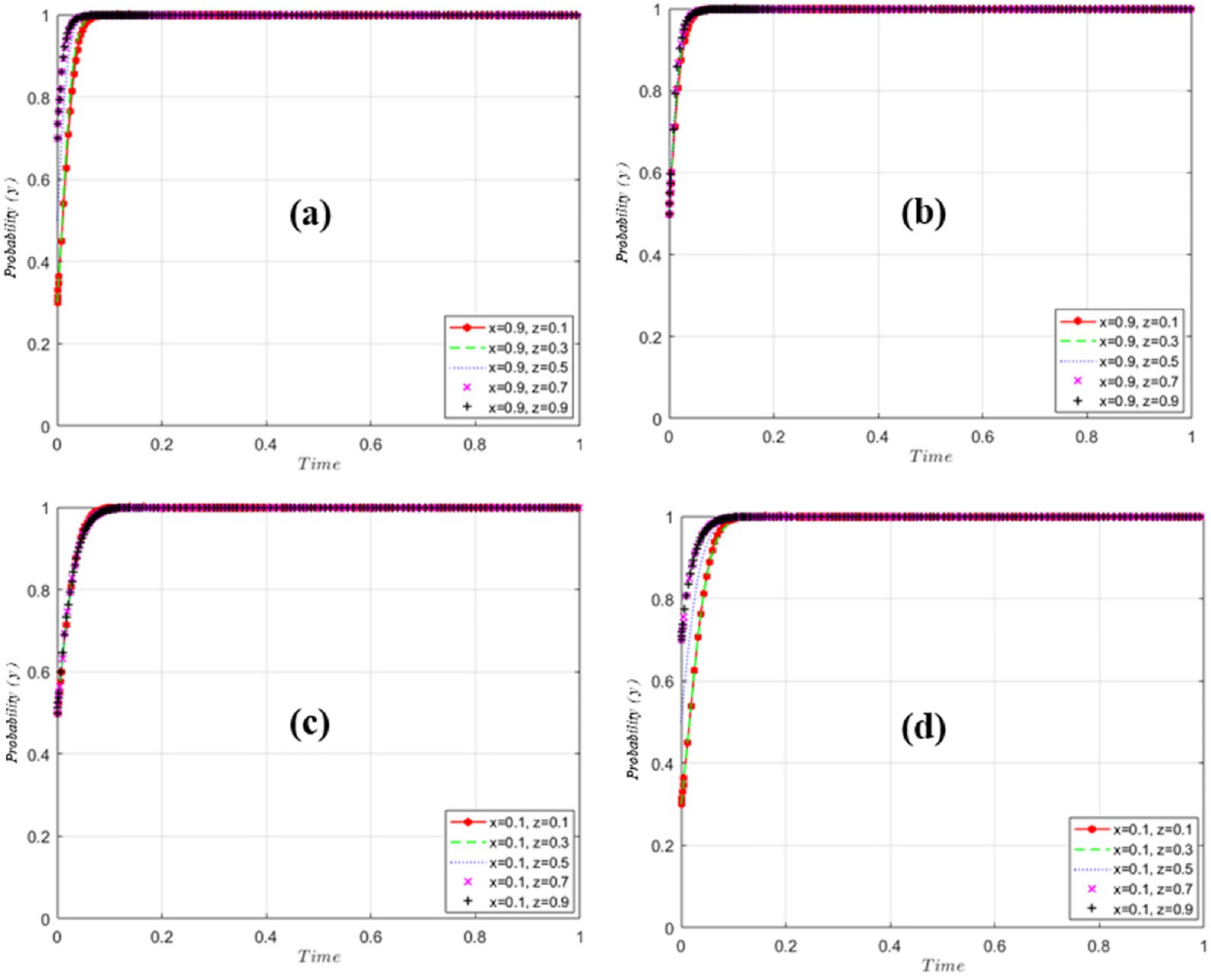
Results of EP execution evolution simulation analysis.

Fig 9 reflects the influence of US and GM strategy choices on EP strategy. When adjusting US and GM strategies, EP eventually stabilizes at probability 1, regardless of the initial probability of "active execution" (Figs 8c and 9a). Adjusting GM and US strategies had little effect on the probability of EP "active execution" (Fig 9b and 9d).

**Fig 10:**
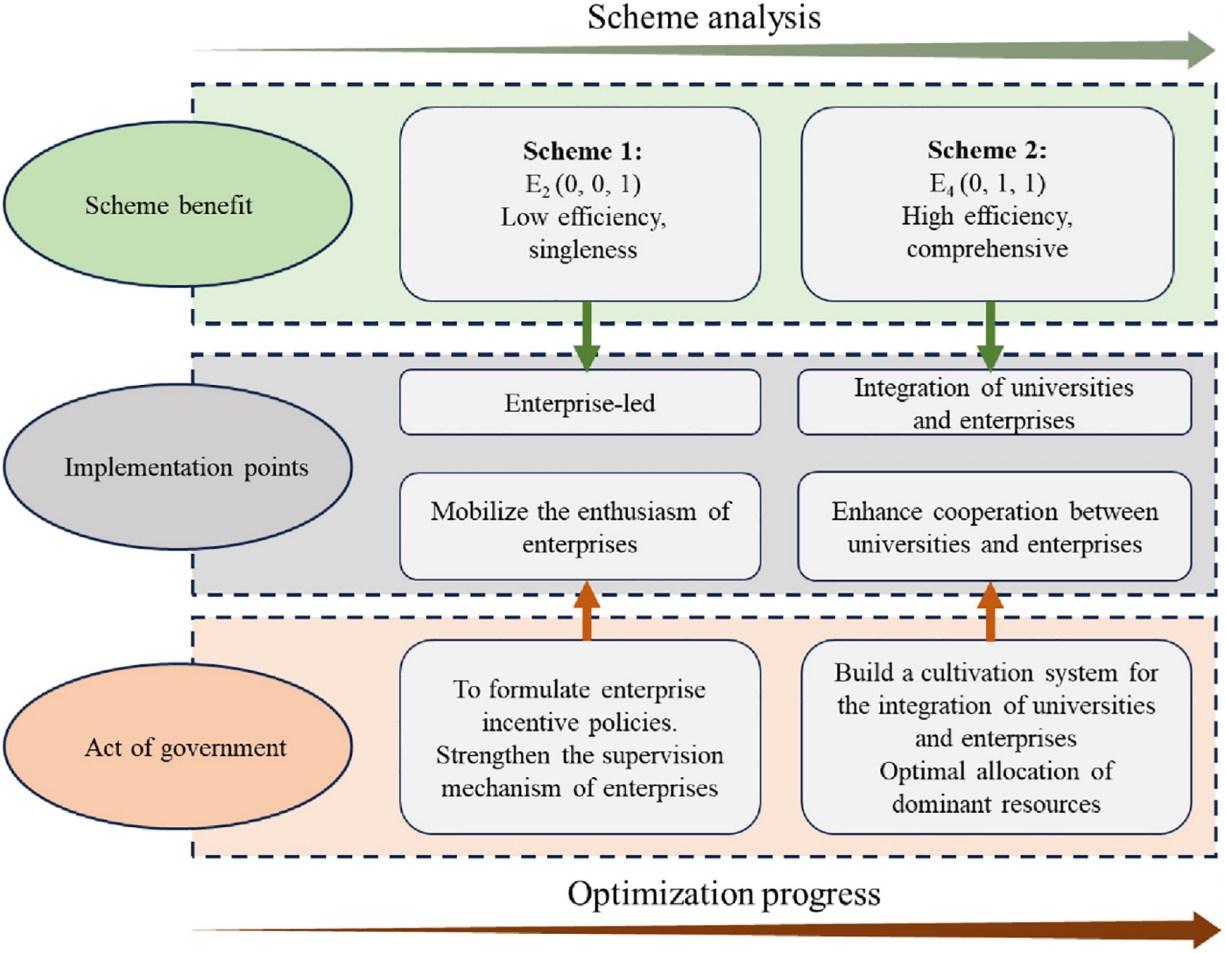
Evolutionary simulation analysis results.

We study the stability of three subjective strategy evolution. and derived E_2_ (0,0,1) and E_4_ (0,1,1) as the ESS of the replicated dynamic system, and analyzed the facilitation mechanism of ETPP under different development scenarios (Fig 10). Scheme 1 exhibits the attributes of homogeneity and low efficiency. In this situation, GM adopts the strategy of non-excitation and US also chooses the strategy of negative implementation, at this time, only EP plays a leading role to ensure the sustainable development of ETPP. However, due to technical and cost issues, EP is not efficient in implementing ETPP and the cost is higher than the benefit. Therefore, the implementation point of this scheme is to mobilize EPs, for which GM should focus on developing effective enterprise incentive policies to increase rewards for enterprises that actively participate in ETPP. Agree that it is also necessary to improve the GM’s monitoring mechanism for EPs. Scheme 2, on the other hand, exhibits composite and high-efficiency attributes. This scheme is characterized by the need for US or EP to adopt active strategies at the same time, and the key core of the implementation of this scheme lies in how to construct and cultivate the integration system of US and EP. the focus of GM’s work is reflected in the optimization of the allocation of resources and the construction of the university-enterprise integration system. The analysis of the scheme shows that the development of ETPP in China is in a period of transition for the optimization from scheme 1 to scheme 2.

## 5 Discuss

The synergistic participation of the three major stakeholders is the optimal path for ETPP in China, in which EP is the actor that pursues the maximization of economic benefits [61]. On the one hand, the inherent contradictory relationship between economic benefits and environmental protection leads EPs to treat ETPP with insufficient enthusiasm [62]. Therefore, it is necessary for the GM to help EPs solve the economic development challenges through policies and subsidies [63]. For example, diversified economic incentives such as tax breaks, financial subsidies, special financial support and loan preferences can fully mobilize Eps [64–66]. On the other hand, in response to EPs’ failure to strictly implement the appointment system for environmental protection positions and even pollution of the environment, GM needs to formulate a complete supervision mechanism.GM has implemented a series of effective supervision systems through the implementation of a series of effective supervision systems [67]. For example, by introducing a special inspection system, an environmental governance information disclosure system, and a public monitoring system, the GM can restrain EP’s speculative behavior in ETPP, thus inhibiting EP’s negative behavior [68, 69]. However, the strategy for US should actively utilize US’s technological R&D and innovation advantages to provide technological and innovation support for EP’s economic development, thus effectively offsetting EP’s incremental costs in ETPP [70, 71]. Eventually, with the gradual improvement of the integration mode, it gradually leads to the ETPP integration system of US and EP, and realizes the optimal allocation of resources.

This paper reveals the interaction mechanism between the participating subjects of ETPP, which has certain theoretical and practical significance. Compared with the previous single research method, this paper innovatively constructs a three-party evolutionary game model of GM, US and EP. The decision-making mechanism of each subject in China’s EPTT is examined. Through theoretical and simulation analyses, key factors of stakeholder strategies are identified and possible evolutionary paths to achieve sustainable development of EPTT are proposed. By applying it to the research and analysis of talent cultivation process, this paper expands the application scope of evolutionary game theory and provides a new methodological strategy for the research of sustainable development of talent cultivation, which is of great practical significance.

## 6 Conclusion and suggestion

In this paper, a three-party evolutionary game model of the three subjects (GM, US and EP) of ETPP is constructed. Eight evolutionary stabilization strategies for the behavioral characteristics and interactions of stakeholders under different scenarios are analyzed, and the optimal strategy stabilization point (0,1,1) is simulated. In addition, this paper explores the influence of several key parameters on each stakeholder’s decision. The main conclusions of this paper are as follows.

1. The comparison of the scenario analysis of the two ESS points shows that only EP plays a dominant role in the (0,0,1) strategy, while US and EP form a fusion mechanism in the (0,1,1) strategy, which jointly promotes the development of ETPP. Considering from the perspective of system stability and benefits, (0,1,1) is the optimized strategy.
2. EPs occupy a more important position in influencing GM decisions. If EP can actively participate in ETPP and choose appropriate strategies, it will achieve satisfactory policy effects. Therefore, GM should play a leading role in the optimal allocation of resources for ETPP and actively promote the construction of an ETPP cultivation system that integrates US and EP.
3. US prefers to reduce the cost of running schools, and the policy incentives given by GM are more conducive to the stability of the system, so GM should reduce the financial support for EPs and increase the policy support, in addition, administrative penalties are not an effective means to improve the motivation of EPs, and GM should adopt a more effective monitoring mechanism to constrain the negative implementation of EPs.

In the ETPP, EP and US assume important implementation roles. Currently, China is in a transition period from purely emphasizing enterprise participation to the integration of US and EP. In the future, China should prioritize the construction of EP participation channels, establish a sound EP participation mechanism, and focus on the introduction of a subsidy policy to encourage EPs to implement ETPP, especially a policy to incentivize green technology innovation. Therefore, it is recommended that a policy of subsidizing environmental technology innovation be introduced. In addition, the environmental information disclosure channel tends to show better results in increasing the level of business participation. An even more effective finding is that the approach of recognizing and rewarding, and thus improving the social image of EPs, would be very helpful in stimulating the potential of firms to participate in ETPPs. However, in order to ensure the quality of ETPPs, GM should also adopt a monitoring approach to constrain the negative strategies of EPs, and the focus of GM’s support for US should be on strengthening the direction of US education, ensuring that the US environmental protection-related specialties, curricula, and knowledge systems not only satisfy national environmental protection goals, but also meet the actual needs of EPs, so as to gain the support and enthusiasm of EPs for their education. GM should also focus on the synergistic effect of ETPP between enterprises and colleges and universities, focusing on promoting the cooperation and interaction between EP and US. In addition, China should make full use of the media, the Internet and public welfare organizations to promote the policy, and make good use of the market mechanism and intangible resources for automatic regulation and policy promotion.

## Supporting information

S1 Dataset (ZIP)
