# Supplementary material for: Sustainable development of environmental protection talents training: Research on the behavior decision of government, university and enterprise under the background of evolutionary game
Source: PLoS One. 2024 Feb 23;19(2):e0298548. doi: 10.1371/journal.pone.0298548 (PMC10890725; doi:10.1371/journal.pone.0298548)
Supplement: S1 Dataset — (ZIP) [file pone.0298548.s001.zip › minimum data set/table.docx]

**Table 1 Variables and meanings related to the three-way game**

| **Interested party** | **Income and expenditure** | **Variable symbol** | **Variable meanings** |
| --- | --- | --- | --- |
| GM | expenditures | O_g1_ | Costs of developing laws and regulations and implementing norms |
|  |  | O_g2_ | EPTT financial outlays to US |
|  |  | O_g3_ | Rewards for EPs when they are "actively performing" |
|  |  | O_g4_ | Rewards for improving overall school performance when US is "actively implemented" |
|  | earnings | I_g1_ | Public image gained by GM when US is "actively implemented" |
|  |  | I_g2_ | Public image gained by GM when EP is "actively implemented" |
|  |  | I_g3_ | Penalization of EP's income in case of EP's "negative enforcement" |
| US | expenditures | O_u1_ | Construction costs of teaching facilities |
|  |  | O_u2_ | US Faculty Training Fees |
|  |  | O_u3_ | Collaborative schooling inputs between US and EPs |
|  |  | α | US expenditure factor |
|  | earnings | O_g2_ | Specialized school support from GM |
|  |  | O_g4_ | Overall US school strength improved and awards received |
|  |  | I_u3_ | Improved overall strength of the US, enhanced reputation, and social benefits gained |
| EP | expenditures | O_e1_ | EP Expenditures for the creation of environmental posts |
|  |  | I_g3_ | Expenditures penalized by the GM in case of EP "Negative Enforcement" |
|  |  | O_e3_ | Expenditures by EP in collaboration with US to provide EPTT with practical teaching conditions |
|  |  | β | EP expenditure factor |
|  | earnings | Ie_1_ | Policy support from GM |
|  |  | O_g3_=I_e2_ | Rewards given by the GM when the EP is "actively executed" |
|  |  | I_e3_ | EP image enhancement, social benefits gained |

**Table 2 Combination of strategies and payoffs of the three-way game**

| **strategy combination** | **GM（x）** | **US（y）** | **EP（z）** |
| --- | --- | --- | --- |
| (Motivation, active implementation, positive enforcement) | I_g1_+I_g2_-O_g1_-O_g2_-O_g3_-O_g4_ | O_g2_+O_g4_+I_u3_-α(O_u1_+O_u2_+O_u3_) | I_e1_+O_g3_+I_e3_ -β(O_e1_+O_e3_) |
| (Incentives, positive implementation, negative enforcement) | I_g1_+I_g3_-O_g1_-O_g2_-O_g4_ | O_g2_+O_g4_+I_u3_-α(O_u1_+O_u2_) | I_e1_-β(O_e1_+O_e3_)-I_g3_ |
| (Incentives, negative implementation, positive implementation) | I_g2_-O_g1_-O_g2_-O_g3_ | O_g2_-α(O_u1_+O_u2_+O_u3_) | I_e1_+O_g3_+I_e3_-β(O_e1_+O_e3_) |
| (Incentives, negative implementation, negative enforcement) | I_g3_-O_g1_-O_g2_ | O_g2_-α(O_u1_+O_u2_) | I_e1_-β(O_e1_+O_e3_)-I_g3_ |
| (No incentives, active implementation, active enforcement) | I_g1_+I_g2_-O_g1_ | I_u3_-α(O_u1_+O_u2_+O_u3_) | I_e3_-β(O_e1_+O_e3_) |
| (No incentives, positive implementation, negative execution) | I_g1_+I_g3_-O_g1_ | I_u3_-α(O_u1_+O_u2_) | -β(O_e1_+O_e3_)-I_e3_ |
| (No incentives, negative implementation, positive implementation) | I_g2_-O_g1_ | 0 | I_e3_-β(O_e1_+O_e3_) |
| (No incentives, negative implementation, negative enforcement) | I_g3_-O_g1_ | -α(O_u1_+O_u2_) | -β(O_e1_+O_e3_)-I_e3_ |

**Table 3 Stability judgment of each equilibrium point**

| Balance points | $\lambda_{1}$ | $\lambda_{2}$ | $\lambda_{3}$ | Stability |
| --- | --- | --- | --- | --- |
| $E_{1}(0,0,0)$ | $I_{e1} (+)$ | $O_{g2} (+)$ | $-O_{g2} (-)$ | Unstable |
| $E_{2}(0,0,1)$ | ${-I}_{e1} (-)$ | $-O_{g2}-O_{g3} (-)$ | $O_{g2}-\alpha(O_{u1}+O_{u2}+O_{u3}) (s)$ | satisfying (a) is ESS |
| $E_{3}(0,1,0)$ | $O_{g2}+O_{g4} (+)$ | $I_{e1}-O_{g3} (s)$ | $-O_{g2} (-)$ | Unstable |
| $E_{4}(0,1,1)$ | $O_{g3}{-I}_{e1} (s)$ | $-O_{g2}-O_{g3}-O_{g4} (-)$ | $\alpha\left( O_{u1}+O_{u2}+O_{u3} \right)-O_{g2} (s)$ | satisfying (b) is ESS |
| $E_{5}(1,0,0)$ | $I_{e1} (+)$ | $O_{g2} (+)$ | $O_{g2}+O_{g4} (+)$ | Unstable |
| $E_{6}(1,0,1)$ | $O_{g2}+O_{g3} (+)$ | $O_{g2}+O_{g4} (+)$ | ${-I}_{e1} (-)$ | Unstable |
| $E_{7}(1,1,0)$ | $I_{e1} (+)$ | $-O_{g2}-O_{g4} (-)$ | $-O_{g2}-O_{g4} (-)$ | Unstable |
| $E_{8}(1,1,1)$ | ${-I}_{e1} (-)$ | $-O_{g2}-O_{g4} (-)$ | $O_{g2}+O_{g3}{+O}_{g4} (+)$ | Unstable |

Notes in the table denotes sign uncertainty; ESS denotes stable strategy; if condition (a and b) is not satisfied, then it is an unstable point. Condition (a): $O_{g2}-\alpha(O_{u1}+O_{u2}+O_{u3})<0$. Condition (b): $O_{g3}{-I}_{e1}<0$ and $\alpha\left( O_{u1}+O_{u2}+O_{u3} \right)-O_{g2}<0$.
